# Report of the Pathogenesis and Pathophysiology of Lyme Disease Subcommittee of the HHS Tick Borne Disease Working Group

**DOI:** 10.3389/fmed.2021.643235

**Published:** 2021-06-07

**Authors:** Sam T. Donta, Leith J. States, Wendy A. Adams, Troy Bankhead, Nicole Baumgarth, Monica E. Embers, Robert B. Lochhead, Brian Stevenson

**Affiliations:** ^1^Falmouth Hospital, Falmouth, MA, United States; ^2^Office of the Assistant Secretary for Health, U.S. Department of Health and Human Services, Washington, DC, United States; ^3^Bay Area Lyme Foundation, Portola Valley, CA, United States; ^4^Department of Veterinary Microbiology and Pathology, Washington State University College of Veterinary Medicine, Pullman, WA, United States; ^5^Center for Immunology and Infectious Diseases, Department of Pathology, Microbiology, and Immunology, School of Veterinary Medicine, University of California, Davis, Davis, CA, United States; ^6^Division of Immunology, Tulane National Primate Research Center, Tulane University School of Medicine, Covington, LA, United States; ^7^Department of Microbiology and Immunology, The Medical College of Wisconsin, Milwaukee, WI, United States; ^8^Department of Microbiology, Immunology, and Molecular Genetics, University of Kentucky College of Medicine, Lexington, KY, United States

**Keywords:** Lyme disease, pathogenesis, pathophysiology, health and human services, tick borne disease working group

## Abstract

An understanding of the pathogenesis and pathophysiology of Lyme disease is key to the ultimate care of patients with Lyme disease. To better understand the various mechanisms underlying the infection caused by *Borrelia burgdorferi*, the Pathogenesis and Pathophysiology of Lyme Disease Subcommittee was formed to review what is currently known about the pathogenesis and pathophysiology of Lyme disease, from its inception, but also especially about its ability to persist in the host. To that end, the authors of this report were assembled to update our knowledge about the infectious process, identify the gaps that exist in our understanding of the process, and provide recommendations as to how to best approach solutions that could lead to a better means to manage patients with persistent Lyme disease.

## Introduction

This Report focuses on the pathogenesis and pathophysiology of Lyme disease. There are other HHS TBDWG subcommittee reports that instead focus on clinical aspects of Lyme disease, and other tick-borne diseases, including issues related to the treatment of these diseases, that are posted on the HHS TBDWG website. Here we summarize presentations by subcommittee members, as well as those of several other, invited investigators. It is recognized that there are many other important contributions by notable investigators in the area of pathogenesis and pathophysiology of Lyme disease and other tick-borne diseases that have not been included here, due to time-limitations for the subcommittee.

## Background

**Figure 1:**
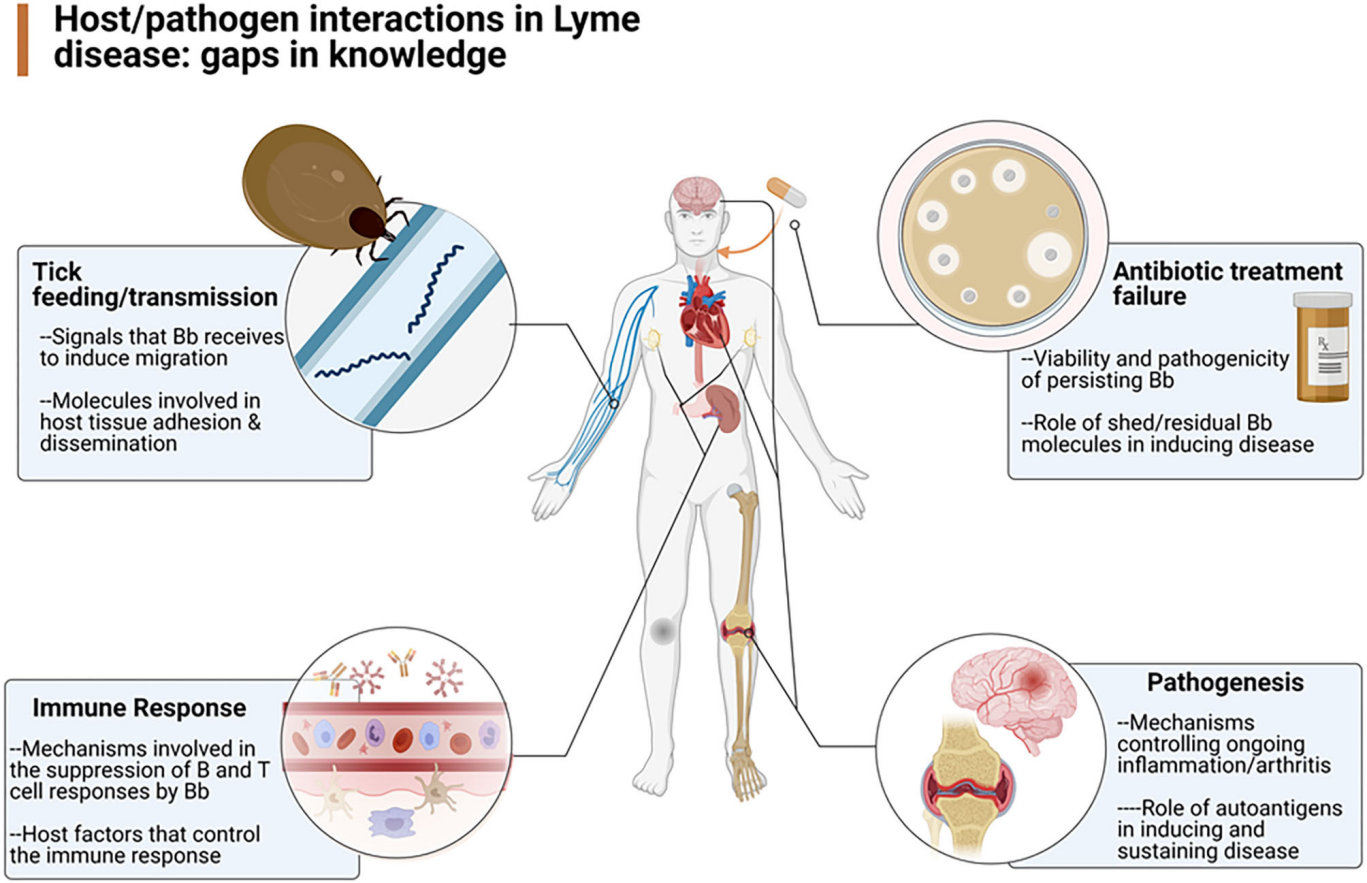
Overview of the gaps in knowledge defined by the working group as areas in need of further research (Created by Biorender.com).

An understanding of the pathogenesis and pathophysiology of Lyme disease is key to the ultimate care of patients with Lyme disease. To better understand the various mechanisms underlying the infection caused by *Borrelia burgdorferi*, the Pathogenesis and Pathophysiology of Lyme Disease Subcommittee was formed to review what is currently known about the pathogenesis and pathophysiology of Lyme disease, from its inception, but also especially about *B. burgdorferi's* ability to persist in the host. To that end, the authors of this report were assembled to update our knowledge about the infectious process, identify the gaps that exist in our understanding of the process (Figure 1), and provide recommendations as to how to best approach solutions that could lead to a better means to manage patients with persistent Lyme disease.

It has been established that the major causative organism of Lyme disease, *B. burgdorferi*, can persist in a number of animal models and human case studies following infection and treatment with a “standard” course of antibiotics (1–4). However, it is still unclear whether human patients with ongoing symptoms associated with Lyme disease continue to have an active infection following completion of what seems as appropriate antibiotic therapy. Thus, the extent to which unresolved infection, incomplete clearance of borrelial antigens, and/or autoimmunity contribute to persistent Lyme disease symptoms is unclear (5, 6).

To better understand the pathogenesis and pathophysiology of Lyme disease, the progression of *B. burgdorferi* from its reservoir in the *Ixodes* tick to transmission into the vertebrate host and to its localization and persistence in neural and other tissues are key steps toward finding means to resolve the infection. The following are descriptions of some of what is known about these various factors of the pathogenesis and pathophysiology of Lyme disease.

### Transmission and Dissemination of *B. burgdorferi* in the Vertebrate Host

In the midgut of a molted, unfed tick, *B. burgdorferi*'s survival in a dormant state requires only a small amount of energy, because little to no bacterial replication occurs (7). Outer surface proteins (Osps) facilitate the pathogen's adhesion to midgut tissue. A tick's ingestion of blood provides *B. burgdorferi* with copious nutrition, resulting in rapid bacterial replication. In turn, *B. burgdorferi* stops producing tick-specific adhesins and starts producing OspC and other factors required for transmission of the pathogen to vertebrates (8). After initiation of a blood meal, the infected tick's midgut swells, and the junctions between midgut cells become thinner. *Borrelia burgdorferi* then penetrates those junctions and enters the tick's salivary glands and salivary ducts, thereby setting the stage for its transmission to a vertebrate *via* tick bite. Upon injection into the vertebrate host, the bacteria adhere to tissues and replicate at the bite site (8, 9). Dissemination of *B. burgdorferi* throughout the vertebrate host involves migration through tissues, as well as transport *via* the bloodstream, resulting in a brief period of bacteremia.

There are a number of questions meriting additional investigation, including processes occurring inside the tick, as well as the processes of initial entry and dissemination, such as the following:

- How does *B. burgdorferi* sense its location in the tick-mammal infectious cycle, then use that information to regulate production of its proteins?
- What are the signals that “tell” *B. burgdorferi* that a vector tick is feeding and that it is time to transmit out of the tick?
- How does *B. burgdorferi* get into the tick's salivary glands and salivary ducts?
- How does *B. burgdorferi* control production of host-specific proteins?
- When bacteria adhere to host tissues at the tick's bite site and then replicate, to what kinds of tissues do they adhere? What types of proteins is *B. burgdorferi* making to facilitate adherence?
- Upon infection of a human, how does *B. burgdorferi* spread? It is known to migrate through skin and other solid tissue, but does it go through the lymphatic system or attach to nerve endings? Does it localize in sensory ganglia? What is the role of adhesins in dissemination throughout the vertebrate host? Are there particular host tissues that attract *B. burgdorferi*?

### Gene Regulation of *B. burgdorferi* During Colonization, Dissemination, and Tissue-Specific Infection in Mice

*Borrelia burgdorferi* can sense whether it is located in a tick or mammal and adapt its response to environmental signals, such as temperature, pH, oxygen levels, carbon dioxide levels, nutrient availability, and reactive oxygen species (7). The rate of bacterial replication has effects on expression levels of numerous infection-associated genes and proteins. Carbon dioxide is important in determining the virulence of *B. burgdorferi* in mice. Borrelial oxidative stress regulator plays a pivotal role in establishing mammalian infection. *B. burgdorferi* can grow and survive without iron; genes generate an oxidative stress response that is involved in the transport of manganese and other metals within *B. burgdorferi*-infected mice. The use of bioluminescent *borrelia* as a tool for studies in mice allows visualization of the kinetics of infection with different strains of the pathogen and enables real-time evaluation of gene expression in the skin, heart, and joints of a mammal infected with *B. burgdorferi*. Notably, localized infection with *B. burgdorferi* becomes more difficult to detect as the pathogen disseminates throughout the mouse. An important gap in knowledge is that it is yet to be determined which genes are required for dissemination of *B. burgdorferi* and its colonization of tissues during later stages of infection (10).

### Role of the Immune System in Response to *B. burgdorferi* Infection

*Borrelia burgdorferi* establishes persistent and non-resolving infections in fully immunocompetent mice, strongly suggesting that the bacteria have developed multiple and likely complex immune evasion strategies (9, 11). Both innate and adaptive immune responses control *B. burgdorferi* in these hosts [reviewed in (12)]. These species rarely, and only transiently, develop clinical manifestations of disease, without an obvious correlation between the tissue-loads of *B. burgdorferi* and clinical manifestations, except in severely immunocompromised mice, for example those that lack T and B cells (SCID mice), or the ability to activate innate immune effectors because of deletions in the toll-like receptor (TLR) 2 or TLR adaptor protein MyD88 (13–15). MyD88-mediated innate immune responses appear to be particularly critical during earliest stages during the establishment of infection (16). Immunoglobulin (Ig) G but not IgM antibodies control *B. burgdorferi* tissue loads, but cannot clear the infection, even when the antibodies are able to passively protect from infection of a new host. IgG acts at least in part through complement-mediated opsonization of the bacteria for subsequent update by macrophage and granulocytes. Data suggest that *B. burgdorferi* suppresses effective innate and adaptive immunity (9, 11); therefore, the immune system is key to understanding persistence of Lyme disease.

B cell responses in these reservoir species are characterized by a lack of continued antibody affinity maturation and the development of long-lived responses due to the rapid collapse of germinal centers. *Borrelia burgdorferi* infection appears to suppress the adaptive immune response, as indicated by the reduced immune response to influenza vaccine in mice infected with *B. burgdorferi* (17). Ongoing work suggests that *B. burgdorferi* also prevents CD4 T cells from mounting an effective immune response to infection, potentially dysregulating effector immune responses in tissues and failing to suppress persistent infection of the host. Data were presented to support the hypothesis that *B. burgdorferi* suppresses and subverts adaptive humoral and cellular immunity to itself and to other antigens. Identifying host immune targets of *Borrelia*-mediated immune suppression might result in the development of approaches that enhance host immunity to this pathogen in a manner similar to strategies that are currently being explored in anti-tumor immunity.

Notably, mice, as reservoir hosts, never clear *B. burgdorferi* infection without antibiotic treatment; humans and non-human primates appear to harbor low-level, persistent *B. burgdorferi* infection as well (18–20). Persistence appears to be a function of active immune suppression and immune evasion tactics. An assay that was developed to detect antibody responses to five antigens of *B. burgdorferi* infection following antibiotic treatment (21) showed that most rhesus macaques infected with *B. burgdorferi* generated responses to most of the antigens, but two showed no specific antibody responses to these antigens (22). In one study in humans, patients who returned to health after antibiotic treatment generated the strongest antibody response (23), reflected by the percentage of plasmablasts that circulated in the blood (24), while those with persisting symptoms had weak responses to antigens or had an anti-oligopeptide permease A2 antibody titer that did not decline. The reasons why some patients develop a good antibody response remain to be determined but might be attributed to host immune factor differences or to differences in the infecting strains of *B. burgdorferi*.

Further studies of immune function in non-human primates previously vaccinated with *B. burgdorferi* found that IgM-producing cells were more frequent and persistent in *B. burgdorferi-*infected primates, results similar to those observed in human patients with persistent Lyme disease as well as in mice. Memory B cells and plasmablasts were reduced in *B. burgdorferi*-infected, unvaccinated macaques compared with vaccinated macaques; whereas CD4 T-cell memory populations appeared similar among groups, activation of T cells was somewhat dampened in the *B. burgdorferi*-infected primates. Areas for future research include determining how long *B. burgdorferi*-induced immune suppression lasts and the impact of persistent infection on effectiveness of vaccines.

### Chemotaxis, Motility, and Immune Evasion as Key Factors in *B. burgdorferi* Spirochete Persistence

Most spirochetes use flagellin proteins as “motors,” with which they move back and forth. This movement can be tracked in real time in mice with the use of multiphoton/confocal microscopy and fluorescently labeled *B. burgdorferi*. Ongoing imaging analysis revealed that the number of spirochetes peaked around 7–10 days after infection (12). This peak was followed by a dramatic drop in spirochete numbers, where they persisted for the duration of the experiment. Spirochetes often tend to reside in the dermis. Of the various resident immune response cells, Langerhans cells were not as effective as macrophages, or other dendritic cells or neutrophils in phagocytosing the bacteria, as spirochetes move up to 80 times faster than any of these immune response cells (12). Neutrophils responded the fastest, but after a certain point, they stop responding, leaving a number of viable spirochetes. There remains a gap in the understanding of the signals involved in this apparent suppression of neutrophil responses. Interleukin 10, the most well-characterized and immunosuppressive cytokine known, is induced early by *B. burgdorferi* to control the innate immune response (25). The innate immune response is important for controlling early infection, independent of the presence of T and B cells. *B. burgdorferi* stimulates several pattern recognition receptors of the innate immune response, inducing pro-inflammatory cytokines (26). Evasion of the innate immune response is accomplished also by multiple complement-binding proteins expressed by *B. burgdorferi* (27), dampening the initial response, as well as IgG-mediated effort functions. Greater understanding is still needed regarding the different roles of the innate and adaptive arms of the immune system in regulating immunity to the spirochetes.

### Role of CD47 and the Immune Response to *B. burgdorferi*

Up-regulation of CD47, a relatively conserved “marker of self,” is a newly discovered mechanism of immune evasion by *B. burgdorferi*. When CD47 binds to signal regulatory protein alpha (SIRP-alpha), there is an inhibition of phagocytosis of those cells, by macrophages. Anti-CD47 antibodies are currently under evaluation in clinical trials for cancer treatment (28, 29). It is hypothesized that *B. burgdorferi* (among other pathogens) can mimic CD47 and thus prevent macrophages from destroying *Borrelia via* phagocytosis (30). Imaging studies of the immune response to *B. burgdorferi* shows that macrophages can send out a “lasso” that wraps around *B. burgdorferi* spirochetes and draws them into the macrophage, usually the first step in the process of phagocytosis. In a few cases, the spirochetes reside in the macrophage but never appear to reach the lysosome, which is where bacterial destruction usually occurs. In donor sera, the addition of the SIRP-alpha binding domains of its receptor CV1G4 *in vitro* can result in increased phagocytosis, presumably by blocking serum-derived SIRP-alpha to CD47-like molecules on the spirochete (31). To understand why the response is more efficient in some settings, the genetic sequences of CD47 and SIRP-alpha were studied showing that SIRP-alpha is highly polymorphic (31). While a number of polymorphisms of CD47 do exist, they are infrequent in humans. Evolutionarily, there has been long-term balancing selection, which ensures that proteins that are vital to the immune response are maintained with maximum diversity, perhaps because the pathogens see some types of SIRP-alpha as beneficial to them. By using mass spectrometry and CV1G4 as a binding partner, a *Borrelia* protein was identified as a CD47-like anti-phagocytic signal. In the absence of this protein, macrophages were more effective in clearing cells. Whether *B. burgdorferi* can survive by inhibiting phagolysosome fusion, as is the case with a number of other known persistent pathogens (32), is currently unknown.

### VlsE Protein-Mediated Immune Evasion

VlsE is a surface-expressed protein able to undergo extensive antigenic variation (33–35). Its expression and ability to undergo antigenic variation is required for *B. burgdorferi* survival and persistence in the presence of a host humoral antibody response targeted against VlsE (36), but also against other surface proteins. A longstanding question has been how *B. burgdorferi* immune escape is accomplished through sequence variation of this single lipoprotein can accomplish immune escape, despite the presence of a substantial number of additional antigens residing on the bacterial surface. A function for VlsE other than its antigenic variation, and thus constant evasion from the humoral antibody response, is not currently known to exist. Although other forms of immune evasion have been proposed, antigenic variation occurs even in antibody-deficient severe combined immunodeficient mice. Among the several models that have been suggested, one scenario proposes that VlsE may act as a shield to obscure the epitopes of other surface antigens (37).

One example of this is the immunogenic Arp protein of *B. burgdorferi*, which is responsible for joint inflammation during infection. Despite Arp eliciting a strong humoral response, antibodies fail to clear the infection. Subsequent studies revealed that VlsE seems to prevent binding of Arp-specific antibodies to the surface of *B. burgdorferi*, thereby providing a possible explanation for the failure of Arp antisera to clear the infection. However, other surface-expressed proteins of *B. burgdorferi* do not seem to be blocked by expression of VlsE, and Arp remains highly immunogenic. Thus, VlsE does not appear to be a universal protector of all *B. burgdorferi* cell surface antigens. Therefore, other, as-of-yet-unknown mechanisms of immune evasion from antibody-mediated *Borrelia* clearance may exist.

### Evidence That Persisting *B. burgdorferi* Are Metabolically Active and Induce Host Gene Expressions

Evidence now exists, from the results of experiments in both murine and non-human primate models, that persisting *B. burgdorferi* can be metabolically active, expressing certain bacterial genes and inducing gene expression changes in the infected host, despite being non-culturable following antibiotic treatment (22, 37–39). In one model, the spirochetes localized to the dura mater of the brain, associated with large-scale changes in gene expression of pro-inflammatory cytokines and chemokines (40, 41). Although there was no evidence of direct infection of the brain itself in this model, certain brain tissues expressed genes related to interferon signaling pathways. Gene expression of other brain functions—for example, glutamate receptors—have not yet been studied. These results, then, provide support for the hypothesis that it is persisting infection that is the cause of persisting symptoms in patients with persistent Lyme disease.

One of the greatest challenges is to actually find means to intervene in the infectious process, especially if no specific markers can be found because of low infectious load or if organisms are in locations other than blood, urine, or cerebrospinal fluid normally used for diagnosis of Lyme disease. Whether different antibiotic regimens can be found to eliminate the persistent state is another challenge that it is hoped can be met with additional targeted research.

### Role of *B. burgdorferi* in the Pathogenesis and Persistence of Lyme Arthritis

*Borrelia burgdorferi* peptidoglycan, the primary component of the bacterial cell wall, has a unique composition and plays an important role in bacterial physiology and host immune responses. *Borrelia burgdorferi* lack the molecular machinery required for recycling of peptidoglycan during cell replication, and the bacteria shed copious amounts of peptidoglycan fragments (42). These fragments are recognized by a host pathogen recognition receptor, NOD2, and cells stimulated with peptidoglycan fragments produce high levels of pro-inflammatory cytokines. Synovial fluid from some human patients with Lyme arthritis, many of whom had received 1–3 months of antibiotic therapy, had high levels of detectible peptidoglycan, as well as anti-peptidoglycan antibodies, despite a lack of any evidence of ongoing infection after antibiotic therapy (42). Thus, it appears that *B. burgdorferi* peptidoglycan might be a persistent antigen in Lyme arthritis (12). Ongoing research is being conducted to determine whether *B. burgdorferi* peptidoglycan plays a role in the pathogenesis and pathophysiology of neuroborreliosis or of persistent Lyme disease other than previously treated Lyme arthritis.

Approximately 60% of untreated individuals with Lyme disease in the United States develop Lyme arthritis. Although most patients with Lyme arthritis respond favorably to 1–3 months of antibiotic therapy, 10–20% of patients have persistent arthritis after treatment (43). A number of genetic and environmental factors contribute to persistent Lyme arthritis, such as infection by certain arthritogenic strains of *B. burgdorferi*, retained spirochetal antigens (for example, peptidoglycans), genetic risk factors, and evidence of prior joint trauma (43, 44). As in rheumatoid arthritis, the prototypical autoimmune joint disease, Lyme arthritis is frequently accompanied by autoimmune T- and B-cell responses to self-antigens (44). These unresolved inflammatory and autoimmune responses may contribute to ongoing arthritis, despite months of antibiotic therapy. Consistent with this hypothesis, nearly all patients with persistent Lyme arthritis experience resolution of arthritis when treated with immunosuppressive drugs, including non-steroidal anti-inflammatory drugs, corticosteroids, and other antirheumatic drugs, such as methotrexate or tumor necrosis factor-alpha inhibitors. Cellular analysis of the arthritic joint has shown that large numbers of IFN-gamma-positive lymphocytes are present in inflamed tissue and surrounding fluid (45). Synovial fibroblasts, the most abundant cell type in synovial tissue, show evidence of immune activation and express major histocompatibility complex (MHC) class II molecules and other immune factors associated with inflammation and lymphocyte activation (44, 45).

Several self-peptides are immunogenic in Lyme disease patients, so there seems to be a breakdown in immune tolerance to self during *B. burgdorferi* infection. Autoimmune B cell responses (but not T cell responses) can be detected early in infection in patients with erythema migrans, but these early autoimmune responses appear to be self-limiting and non-pathogenic. T cell autoimmunity accompanies B cell autoimmunity later in disease, such as during Lyme arthritis. In late-stage disease, Lyme-disease-associated autoantibodies correlate with clinical features of arthritis, suggesting that autoimmunity in Lyme disease may become pathogenic over time. Lyme arthritis progresses from early invasion of synovial tissue to early inflammatory responses to later inflammatory responses, and then to late tissue repair and wound healing (44, 45). The role of infection as an autoimmune trigger in Lyme disease is poorly understood, leading to the following questions:

- What are the mechanisms by which *B. burgdorferi* infection causes ongoing arthritic joint disease in a subset of patients?
- Are ongoing disease symptoms caused by the presence of *Borrelia* antigens (such as peptidoglycans) rather than active infection and, if so, why are they not cleared from the host?
- Does *Borrelia* infection trigger autoimmune responses in infected individuals and are these autoimmune responses pathogenic in some patients?

Questions also remain regarding the role of immunosuppressive treatments vs. differing antibiotic treatment regimens for persistent Lyme arthritis, if peptidoglycan is an inflammatory agent and persists despite 1–3 months of antibiotic therapy. Patients who have persistent Lyme arthritis may represent a different condition than do people with other Lyme disease syndromes.

Whereas, prompt treatment of early Lyme disease, using antibiotics with differing mechanisms of action, is usually effective in prevention of persistence of *B. burgdorferi* and persistent Lyme disease, similar antibiotic treatments for persistent *B. burgdorferi* in animal models and in patients with persistent Lyme disease appear to be ineffective. The reasons for this difference are unclear, but may be due to a number of possible mechanisms:

- The bacteria may be dormant or incapable of replication, yet there may be the presence of residual antigens or the periodic release of antigens, to which the host responds to produce the symptoms associated with persistent Lyme disease.
- The bacteria may be entrenched in areas either inaccessible to certain classes of antibiotics (for example, poorly vascularized connective tissue, intracellular compartments), or higher doses of antibiotics are needed to achieve levels that impede metabolic activity.
- The bacteria may become antibiotic-tolerant, requiring repeated courses of antibiotic treatment, combinations of antibiotics, or periods of treatment alternating with periods of no treatment.

There are indications that certain treatment regimens (for example, tetracycline instead of doxycycline, the combination of a macrolide antibiotic and an alkalinizing agent) are effective in treating the persistent state if given over longer durations of time rather than the usual 2–4-week periods. There is ongoing research as well, some in the discovery phase, using novel compounds to treat persisting organisms. There is also some indication that the intestinal microbiota may play an important role in the persistence or ability to eradicate persisting organisms.

## Summary

The results of studies into the pathogenesis and pathophysiology of Lyme disease, with the focus on the persistent state of the causative organism, *B. burgdorferi*, have begun to elucidate the mechanisms underlying the process by which the persistent state occurs. However, important gaps exist into how the process develops, from the organism's existence in the *Ixodes* tick, to its entry into the host, to its effects on the immune system, to its distribution and ability to persist in certain tissues, to its ability to persist despite innate and other host immune system responses, and to its ability to persist despite certain antibiotic treatments. But there is reason for optimism that additional research into the pathogenetic and pathophysiologic mechanisms will lead to a better understanding of the processes involved and ultimately to a better means of preventing and treating patients with persistent Lyme disease.

## Author's Note

This report is a condensed version of the full report that appears on the HHS TBDWG website, and has the permission of the HHS designated officer assigned to this working group.

## Author Contributions

All authors contributed to the discussion and writing of the Report.

## Conflict of Interest

The authors declare that the research was conducted in the absence of any commercial or financial relationships that could be construed as a potential conflict of interest.

## References

[1] Bockenstedt LK Gonzalez DG Haberman AM Belperron AA. Spirochete antigens persist near cartilage after murine Lyme borreliosis therapy. J Clin Invest. (2012) 122:2652–60. 10.1172/JCI58813 22728937 PMC3386809

[2] Embers ME Barthold SW Borda JT Bowers L Doyle L Hodzic E. Persistence of *Borrelia burgdorferi* in rhesus macaques following antibiotic treatment of disseminated infection. PLoS ONE. (2012) 7:e29914. 10.1371/journal.pone.0029914 22253822 PMC3256191

[3] Sapi E Kasliwala RS Ismail H Torres JP Oldakowski M Markland S. The Long-term persistence of *Borrelia burgdorferi* antigens and DNA in the tissues of a patient with Lyme disease. Antibiotics (Basel). (2019) 8:183. 10.3390/antibiotics8040183 31614557 PMC6963883

[4] Strle F Cheng Y Cimperman J Maraspin V Lotric-Furlan S Nelson JA. Persistence of *Borrelia burgdorferi sensu lato* in resolved erythema migrans lesions. Clin Infect Dis. (1995) 21:380–9. 10.1093/clinids/21.2.380 8562748

[5] Marques A. Chronic Lyme disease: a review. Infect Dis Clin North Am. (2008) 22:341–60, vii-viii. 10.1016/j.idc.2007.12.011 18452806 PMC2430045

[6] Kullberg BJ Vrijmoeth HD van de Schoor F Hovius JW. Lyme borreliosis: diagnosis and management. BMJ. (2020) 369:m1041. 10.1136/bmj.m1041 32457042

[7] Stevenson B Seshu J. Regulation of gene and protein expression in the Lyme disease spirochete. Curr Top Microbiol Immunol. (2018) 415:83–112. 10.1007/82_2017_49 29064060

[8] Jutras BL Chenail AM Stevenson B. Changes in bacterial growth rate govern expression of the *Borrelia burgdorferi* OspC and Erp infection-associated surface proteins. J Bacteriol. (2013) 195:757–64. 10.1128/JB.01956-12 23222718 PMC3562092

[9] Radolf JD Caimano MJ Stevenson B Hu LT. Of ticks, mice and men: understanding the dual-host lifestyle of Lyme disease spirochaetes. Nat Rev Microbiol. (2012) 10:87–99. 10.1038/nrmicro2714 22230951 PMC3313462

[10] Arnold WK Savage CR Brissette CA Seshu J Livny J Stevenson B. RNA-Seq of *Borrelia burgdorferi* in multiple phases of growth reveals insights into the dynamics of gene expression, transcriptome architecture, and noncoding RNAs. PLoS ONE. (2016) 11:e0164165. 10.1371/journal.pone.0164165 27706236 PMC5051733

[11] Tracy KE Baumgarth N. *Borrelia burgdorferi* manipulates innate and adaptive immunity to establish persistence in rodent reservoir hosts. Front Immunol. (2017) 8:116. 10.3389/fimmu.2017.00116 28265270 PMC5316537

[12] Bockenstedt LK Wooten RM Baumgarth N. Immune response to *Borrelia*: lessons from Lyme disease spirochetes. Curr Issues Mol Biol. (2021) 42:145–90. 10.21775/cimb.042.145 33289684 PMC10842262

[13] Wooten RM Ma Y Yoder RA Brown JP Weis JH Zachary JF. Toll-like receptor 2 plays a pivotal role in host defense and inflammatory response to *Borrelia burgdorferi*. Vector Borne Zoonotic Dis. (2002) 2:275–8. 10.1089/153036602321653860 12804169

[14] Bolz DD Sundsbak RS Ma Y Akira S Kirschning CJ Zachary JF. MyD88 plays a unique role in host defense but not arthritis development in Lyme disease. J Immunol. (2004) 173:2003–10. 10.4049/jimmunol.173.3.2003 15265935

[15] Behera AK Hildebrand E Bronson RT Perides G Uematsu S Akira S. MyD88 deficiency results in tissue-specific changes in cytokine induction and inflammation in interleukin-18-independent mice infected with *Borrelia burgdorferi*. Infect Immun. (2006) 74:1462–70. 10.1128/IAI.74.3.1462-1470.2006 16495516 PMC1418660

[16] Troy EB Lin T Gao L Lazinski DW Camilli A Norris SJ. Understanding barriers to *Borrelia burgdorferi* dissemination during infection using massively parallel sequencing. Infect Immun. (2013) 81:2347–57. 10.1128/IAI.00266-13 23608706 PMC3697624

[17] Elsner RA Hastey CJ Olsen KJ Baumgarth N. Suppression of long-lived humoral immunity following *Borrelia burgdorferi* infection. PLoS Pathog. (2015) 11:e1004976. 10.1371/journal.ppat.1004976 26136236 PMC4489802

[18] Crossland NA Alvarez X Embers ME. Late disseminated Lyme disease: associated pathology and spirochete persistence posttreatment in rhesus macaques. Am J Pathol. (2018) 188:672–82. 10.1016/j.ajpath.2017.11.005 29242055 PMC5840488

[19] Coulter P Lema C Flayhart D Linhardt AS Aucott JN Auwaerter PG. Two-year evaluation of *Borrelia burgdorferi* culture and supplemental tests for definitive diagnosis of Lyme disease. J Clin Microbiol. (2005) 43:5080–4. 10.1128/JCM.43.10.5080-5084.2005 16207966 PMC1248466

[20] Pachner AR Zhang WF Schaefer H Schaefer S O'Neill T. Detection of active infection in nonhuman primates with Lyme neuroborreliosis: comparison of PCR, culture, and a bioassay. J Clin Microbiol. (1998) 36:3243–7. 10.1128/JCM.36.11.3243-3247.1998 9774573 PMC105309

[21] Embers ME Hasenkampf NR Barnes MB Didier ES Philipp MT Tardo AC. Five-antigen fluorescent bead-based assay for diagnosis of Lyme disease. Clin Vaccine Immunol. (2016) 23:294–303. 10.1128/CVI.00685-15 26843487 PMC4820514

[22] Embers ME Hasenkampf NR Jacobs MB Tardo AC Doyle-Meyers LA Philipp MT. Variable manifestations, diverse seroreactivity and post-treatment persistence in non-human primates exposed to *Borrelia burgdorferi* by tick feeding. PLoS ONE. (2017) 12:e0189071. 10.1371/journal.pone.0189071 29236732 PMC5728523

[23] Aucott JN Crowder LA Kortte KB. Development of a foundation for a case definition of post-treatment Lyme disease syndrome. Int J Infect Dis. (2013) 17:e443–9. 10.1016/j.ijid.2013.01.008 23462300

[24] Blum LK Adamska JZ Martin DS Rebman AW Elliott SE Cao RRL. Robust B cell responses predict rapid resolution of Lyme disease. Front Immunol. (2018) 9:1634. 10.3389/fimmu.2018.01634 30072990 PMC6060717

[25] Brown JP Zachary JF Teuscher C Weis JJ Wooten RM. Dual role of interleukin-10 in murine Lyme disease: regulation of arthritis severity and host defense. Infect Immun. (1999) 67:5142–50. 10.1128/IAI.67.10.5142-5150.1999 10496888 PMC96863

[26] Oosting M Buffen K van der Meer JW Netea MG Joosten LA. Innate immunity networks during infection with *Borrelia burgdorferi*. Crit Rev Microbiol. (2016) 42:233–44. 10.3109/1040841X.2014.929563 24963691

[27] Kraiczy P. Hide and seek: how Lyme disease spirochetes overcome complement attack. Front Immunol. (2016) 7:385. 10.3389/fimmu.2016.00385 27725820 PMC5036304

[28] van der Burg SH Arens R Melief CJ. Immunotherapy for persistent viral infections and associated disease. Trends Immunol. (2011) 32:97–103. 10.1016/j.it.2010.12.006 21227751

[29] Willingham SB Volkmer JP Gentles AJ Sahoo D Dalerba P Mitra SS. The CD47-signal regulatory protein alpha (SIRPa) interaction is a therapeutic target for human solid tumors. Proc Natl Acad Sci U S A. (2012) 109:6662–7. 10.1073/pnas.1121623109 22451913 PMC3340046

[30] Tal MC Torrez Dulgeroff LB Myers L Cham LB Mayer-Barber KD Bohrer AC. Upregulation of CD47 Is a host checkpoint response to pathogen recognition. mBio. (2020) 11:mBio.e-1293-20. 10.1128/mBio.01293-20 32576678 PMC7315125

[31] Weiskopf K Ring AM Ho CC Volkmer JP Levin AM Volkmer AK. Engineered SIRPalpha variants as immunotherapeutic adjuvants to anticancer antibodies. Science. (2013) 341:88–91. 10.1126/science.1238856 23722425 PMC3810306

[32] Donta ST. Macrolide therapy of chronic Lyme disease. Med Sci Monit. (2003) 9:PI136–142. 14586290

[33] Bankhead T Chaconas G. The role of VlsE antigenic variation in the Lyme disease spirochete: persistence through a mechanism that differs from other pathogens. Mol Microbiol. (2007) 65:1547–58. 10.1111/j.1365-2958.2007.05895.x 17714442

[34] Zhang JR Norris SJ. Genetic variation of the *Borrelia burgdorferi* gene vlsE involves cassette-specific, segmental gene conversion. Infect Immun. (1998) 66:3698–704. 10.1128/IAI.66.8.3698-3704.1998 9673251 PMC108404

[35] Zhang JR Norris SJ. Kinetics and *in vivo* induction of genetic variation of vlsE in *Borrelia burgdorferi*. Infect Immun. (1998) 66:3689–97. 10.1128/IAI.66.8.3689-3697.1998 9673250 PMC108403

[36] Bankhead T. Role of the VlsE lipoprotein in immune avoidance by the lyme disease spirochete *Borrelia burgdorferi*. For Immunopathol Dis Therap. (2016) 7:191–204. 10.1615/ForumImmunDisTher.2017019625 29876140 PMC5986088

[37] Greenmyer JR Gaultney RA Brissette CA Watt JA. Primary human microglia are phagocytically active and respond to *Borrelia burgdorferi* with upregulation of chemokines and cytokines. Front Microbiol. (2018) 9:811. 10.3389/fmicb.2018.00811 29922241 PMC5996889

[38] Caskey JR Hasenkampf NR Martin DS Chouljenko VN Subramanian R Cheslock MA. The functional and molecular effects of doxycycline treatment on *Borrelia burgdorferi* phenotype. Front Microbiol. (2019) 10:690. 10.3389/fmicb.2019.00690 31057493 PMC6482230

[39] Hodzic E Imai D Feng S Barthold SW. Resurgence of persisting non-cultivable *Borrelia burgdorferi* following antibiotic treatment in mice. PLoS ONE. (2014) 9:e86907. 10.1371/journal.pone.0086907 24466286 PMC3900665

[40] Casselli T Divan A Vomhof-DeKrey EE Tourand Y Pecoraro HL Brissette CA. A murine model of Lyme disease demonstrates that *Borrelia burgdorferi* colonizes the dura mater and induces inflammation in the central nervous system. PLoS Pathog. (2021) 17:e1009256. 10.1371/journal.ppat.1009256 33524035 PMC7877756

[41] Divan A Casselli T Narayanan SA Mukherjee S Zawieja DC Watt JA. *Borrelia burgdorferi* adhere to blood vessels in the dura mater and are associated with increased meningeal T cells during murine disseminated borreliosis. PLoS ONE. (2018) 13:e0196893. 10.1371/journal.pone.0196893 29723263 PMC5933741

[42] Jutras BL Lochhead RB Kloos ZA Biboy J Strle K Booth CJ. *Borrelia burgdorferi* peptidoglycan is a persistent antigen in patients with Lyme arthritis. Proc Natl Acad Sci U S A. (2019) 116:13498–507. 10.1073/pnas.1904170116 31209025 PMC6613144

[43] Arvikar SL Steere AC. Diagnosis and treatment of Lyme arthritis. Infect Dis Clin North Am. (2015) 29:269–80. 10.1016/j.idc.2015.02.004 25999223 PMC4443866

[44] Lochhead RB Arvikar SL Aversa JM Sadreyev RI Strle K Steere AC. Robust interferon signature and suppressed tissue repair gene expression in synovial tissue from patients with postinfectious, *Borrelia burgdorferi*-induced Lyme arthritis. Cell Microbiol. (2019) 21:e12954. 10.1111/cmi.12954 30218476 PMC6724218

[45] Lochhead RB Ordonez D Arvikar SL Aversa JM Oh LS Heyworth B. Interferon-gamma production in Lyme arthritis synovial tissue promotes differentiation of fibroblast-like synoviocytes into immune effector cells. Cell Microbiol. (2019) 21:e12992. 10.1111/cmi.12992 30550623 PMC6336510

